# Importance of correctly interpreting magnetic resonance imaging to diagnose posterior reversible encephalopathy syndrome associated with HELLP syndrome: a case report

**DOI:** 10.1186/s12880-017-0208-6

**Published:** 2017-05-25

**Authors:** Syuichi Tetsuka, Hiroaki Nonaka

**Affiliations:** 1 Department of Neurology, Hospital of Yuki, 9629-1, Yuki, Yuki-City, Ibaraki 307-0001 Japan; 2 Obstetrics & Gynecology, Hospital of International University of Health and Welfare, 537-3, Iguchi, Nasushiobara, Tochigi 329-2763 Japan

**Keywords:** HELLP syndrome, Posterior reversible encephalopathy syndrome, Magnetic resonance imaging

## Abstract

**Background:**

Severe haemolysis, elevated liver enzyme levels, and low platelet count (HELLP) syndrome in pregnancy are possible underlying trigger factors for posterior reversible encephalopathy syndrome (PRES). Magnetic resonance imaging (MRI) shows diffuse signal abnormalities involving the subcortical white matter in the parieto-occipital lobes. Although the diagnosis of RPES was clearly established by the distinctive reversibility of clinical and radiological abnormalities, it is difficult to distinguish from differential diagnosis. Thus, it is important to correctly interpret MRI.

**Case presentation:**

We describe a case of HELLP syndrome with PRES. A 38-year-old pregnant woman was admitted to our hospital as an emergency case with a complaint of upper abdominal pain and headache at 29 weeks of pregnancy and the development of HELLP syndrome. An emergency caesarean section was immediately performed. After the operation, the patient received intravenous corticosteroids, and her blood pressure was controlled. Thereafter, she showed an altered mental status. MRI showed hypersignal intense lesions in the cortical and subcortical white matter in the occipital lobes, basal ganglia and callosal splenium in both the fluid-attenuated inversion recovery (FLAIR) sequence and apparent diffusion coefficient (ADC), but these lesions were not recognized in diffusion-weighted imaging (DWI). These images were suggestive of PRES. The patient was kept in the hospital and received the appropriate treatment, after which the patient’s level of consciousness improved and all laboratory tests and imaging examinations returned normal.

**Conclusion:**

The MRI findings were useful for the prompt diagnosis of PRES, characterized by hypersignals in FLAIR and ADC, but not in DWI. Additionally, there was an “atypical” MRI appearance of basal ganglial and callosal splenial involvement in this case, which may mistakenly lead clinicians to diagnose other aetiologies than typical PRES. It is considered that vasogenic oedema is the main pathology of PRES according to the MRI image findings. MRI is the gold standard for diagnosing PRES because it can provide information about cerebral involvement earlier than CT; further, it can be a useful tool in the differential diagnosis. This technique facilitated the prompt diagnosis and treatment of the said patient, ultimately resulting in a good outcome.

## Background

Haemolysis, elevated liver enzymes, and low platelet count (HELLP) syndrome is a complication of severe forms of pre-eclampsia that compromises the blood system with haemolysis, hepatic lesions, and low platelet counts and can cause a lethal condition with a high mortality rate of up to 25% [1]. Along with maternal death, HELLP syndrome is associated with the development of focal neurological complications, including seizure, ischaemic and haemorrhagic strokes, cerebral venous thrombosis, eclampsia, cerebral artery dissection, encephalitis, and posterior reversible encephalopathy syndrome (PRES).

PRES is a clinical condition characterized by temporary neurological symptoms such as acute headache, altered mental status, vision loss, and coma. The prognosis of PRES is good when an appropriate treatment is received; symptoms generally improve in approximately 3–8 days, and imaging abnormalities disappear within a few weeks. However, 5%–12% of such cases can assume a negative course with irreversible brain damage culminating in persistent severe neurological deficit or death [2, 3]. Early diagnosis and prompt treatment of PRES are essential to avoid irreversible neurological deficits. The neuroradiographic findings of PRES by magnetic resonance imaging (MRI) are symmetrical subcortical white matter or cortical oedema in the occipital and parietal lobes, brain stem, basal ganglia, frontal lobes, and cerebellum [4, 5]. The typical manifestations of PRES are MRI abnormalities that occur primarily in the subcortical white matter and often extend to the cortical surface, sparing the deep white matter in the classic posterior parietal or occipital lobe region most consistently. The “atypical” findings of basal ganglial and callosal splenial involvement on MRI may mistakenly lead clinicians to diagnose other aetiologies, such as stroke, hypoxic-ischaemic injury, or an overdose of pain medication (e.g., opioids). Intravenous contrast administration is not indicated to diagnose PRES because contrast enhancement is reported to have a variable incidence [6]. Thus, plain MRI is essential in the diagnosis of PRES. We report a case of PRES that showed atypical findings of basal ganglial and callosal splenial involvement on MRI in the setting of severe HELLP syndrome that improved without sequelae because of prompt diagnosis and treatment.

## Case presentation

A 38-year-old primigravid reported for a pregnancy screening after her normal gestation period of 28 weeks without hypertension and a normal blood and urine test. She had not experienced any problems during her pregnancy until then. However, later, she was admitted to our hospital of International University of Health and Welfare as an emergency case with a complaint of upper abdominal pain and headache at 29 weeks of pregnancy. On arrival, her Glasgow Coma Scale (GCS) was E4V5M6. Her blood pressure at admission was 200/100 mmHg. Her blood biochemistry showed hypertransaminasemia with aspartate aminotransferase (AST) at 821 IU/L, elevated lactate dehydrogenase (LDH) at 1895 IU/L, and a low platelet count at 4.3 × 10^5^/μL. The patient’s serum bilirubin level was 1.7 mg/dl. Glucose, creatinine (Cr), and serum electrolytes were normal. According to HELLP syndrome for the diagnostic criteria (Table 1) [7], she was diagnosed with pregnancy–HELLP syndrome and was immediately scheduled for an emergency caesarean section. The new-born male weighed 975 g with an Apgar score of 7 at the first minute and 8 at the fifth minute. After the operation, the patient was administered intravenous corticosteroids, and nifedipine was continued to maintain her blood pressure below approximately 150/90 mmHg. Gabexate mesilate and anti-thrombin III were also infused to treat intravascular coagulation, which is often observed in patients with HELLP syndrome.

**Table 1:** The diagnostic criteria and classification of HELLP syndrome [7]

Class 1: requires severe thrombocytopenia (platelets ≤50,000/μl), evidence of hepatic dysfunction (AST [aspartate aminotransferase] and/or ALT [alanine aminotransferase] ≥70 IU/l), and evidence suggestive of hemolysis (total serum LDH [lactate dehydrogenase] ≥600 IU/l).	
Class 2: requires similar criteria except thrombocytopenia is moderate (>50,000 to ≤100,000/μl).	
Class 3: includes patients with mild thrombocytopenia (platelets >100,000 but ≤150,000/μl), mild hepatic dysfunction (AST and/or ALT ≥40 IU/l), and hemolysis (total serum LDH ≥600 IU/L).	

**Fig. 1:**
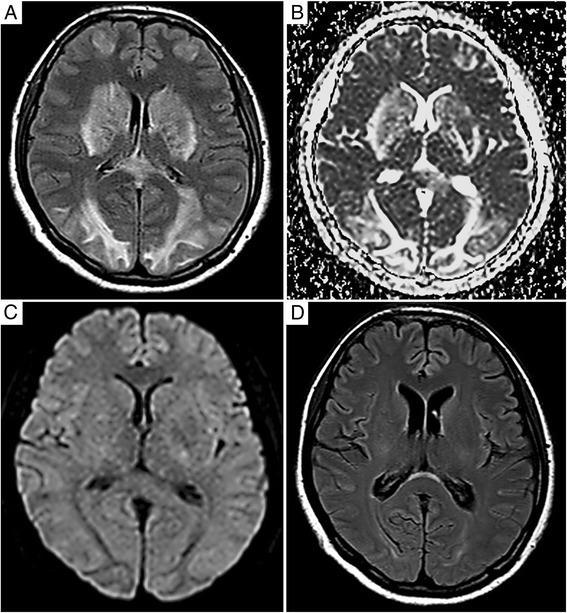
MRI showed hypersignal intense lesions in the cortical and subcortical white matter in the occipital lobes, basal ganglia and callosal splenium in both the fluid-attenuated inversion recovery (FLAIR) sequence (**a**) and apparent diffusion coefficient (ADC) (**b**), but these lesions were not recognized in diffusion-weighted imaging (DWI) (**c**). (**b**) The follow-up brain MRI, performed 2 weeks later, showed complete resolution of the lesions

On the second postoperative day, she was assessed by a neurologist. She was conscious but very drowsy and could move her limbs and follow simple orders, and her GCS was E3V4M5. However, she occasionally presented with delirium, which is characterized by a fluctuating mental status. A brain MRI was immediately performed because of her drowsy and confused state on the neurological examination. MRI showed hypersignal intense lesions in the cortical and subcortical white matter in the occipital lobes, basal ganglia and callosal splenium in both the fluid-attenuated inversion recovery (FLAIR) sequence (Fig. 1a) and apparent diffusion coefficient (ADC) (Fig. 1b), but these lesions were not recognized in diffusion-weighted imaging (DWI) (Fig. 1c). DWI was normal. These images were suggestive of PRES. The aforementioned treatment was continued. On biochemical analysis, it was observed that the liver function improved (AST 59 IU/L, LDH 932 IU/L); however, renal impairment deteriorated further (Cr 4.27 mg/dl). In addition, her urine volume decreased to approximately 400 ml/day. Subsequently, she received haemodialysis 4 times starting on the 5^th^ postoperative day.

Subsequently, patient’s level of consciousness improved, and her renal functional also gradually improved. A complete resolution of the lesions (Fig. 1d) was observed on the follow-up brain MRI performed in 2 weeks after caesarean section. The patient was kept in the hospital for 21 days, and throughout this period, all laboratory tests and imaging examinations returned to normal. The patient was then released, and outpatient follow-up was continued.

## Discussion

We present a case of HELLP syndrome that occurred during pregnancy associated with clinical and neuroradiological findings consistent with PRES. The major complications in such cases are eclampsia, haematologic (intravascular disseminated coagulation) and cardiopulmonary (pulmonary oedema and acute cardiac insufficiency) events, and neurological alterations (cerebral bleeding, hypoxic–ischaemic encephalopathy, eclampsia). When HELLP syndrome complicates eclampsia, the maternal and perinatal death rates increase with delayed diagnosis or inappropriate treatment, accounting for the poor overall prognosis [8]. Early treatment reduces both maternal and perinatal complications. Headache, altered consciousness, seizures, and visual symptoms, including cortical blindness, are frequent clinical findings associated with PRES.

PRES is predominantly characterized by reversible brain oedema affecting the occipital and posterior parietal regions and is usually described in association with hypertensive encephalopathy, eclampsia, and renal failure [4, 5]. A mild elevation in blood pressure may occur with PRES, even in patients without hypertension [4, 9]. We hypothesize that because blood pressure has a tendency to increase during pregnancy, a physiological state connected to PRES may be involved in the development of this case. However, PRES commonly occurs in patients with eclampsia. Cerebral vasospasm is the proposed aetiology of eclampsia, although the patient did not show eclampsia, which suggests that vasogenic oedema of the brain plays a major pathophysiological role in most patients with PRES. However, PRES is not always reversible. McKinney et al. [10] reported that DWI abnormalities were found in a minority (15–20%), which later studies have shown to potentially lead to neurologic sequelae and encephalomalacia. The lack of DWI abnormalities in this patient solidified the diagnosis of PRES. However, this patient had basal ganglial abnormalities, which could confound the diagnosis described in 10–15% of the patients in the aforementioned study [10]. Considering these factors, this PRES case was relatively rare.

Vasogenic oedema due to cerebrovascular autoregulatory dysfunction results in the leakage of fluid into the interstitium. It was considered that vasogenic oedema is the main pathology of PRES by MRI imaging findings. Endogenous-exogenous factors injure the endothelial cells in the brain blood vessels in PRES. Furthermore, ischaemia due to vasospasm accelerates endothelial injury. If there is an increase in blood pressure due to whole-body vasospasm and hypertension, even if it is slight, it is considered that the autoregulatory capacity of the blood–brain barrier and cerebral blood flow collapses immediately. Therefore, it is presumed that vasogenic oedema easily occurs. The reason that PRES often develops with an increase in blood pressure is thought to involve an increase in blood pressure accompanying vasospasm and injury of vascular endothelial cells, including ischaemia. More recent evidence suggests that endothelial injury is a more likely cause, as many (up to 50%) PRES patients do not have elevated BP. However, BP and other factors certainly contribute. Additionally, the fact that DWI is positive suggests that cytotoxic oedema can coexist or occur superimposed in a minority and that vasogenic oedema is just one component of the injury. It has become clear that perhaps hypoperfusion or hyperperfusion alone are inadequate explanations for the manifestations of PRES. A more novel theory is that of a systemic toxicity, perhaps with increased leukocyte trafficking, which results in endothelial dysfunction. Hypoperfusion and vasoconstriction may lead to hypoxia with the upregulation of vascular endothelial growth factor; as a result, endothelial permeability increases. This process may be modulated by changes in blood pressure, with increased autoregulatory vasoconstriction in response to increases in blood pressure. In summary, 1) severe hypertension leads to failed auto-regulation, subsequent hyperperfusion, and endothelial injury/vasogenic oedema; and 2) vasoconstriction and hypoperfusion lead to brain ischaemia and subsequent vasogenic oedema [11, 12]. This is important when considering the MRI findings of PRES. Both vasogenic and cytotoxic oedema show signal hyperintensity on FLAIR. However, DWI only shows markedly high density for cytotoxic oedema. In addition, ADC maps only display the diffusion component and are hyperintense in PRES, in contrast to being hypointense in cytotoxic oedema that causes ischaemia (cerebral infraction) (Table 2). Such MRI findings were also recognized in this case. In addition, there are characteristic differences on brain lesions, indicating abnormal MRI imaging between PRES patients unrelated to pregnancy and those with HELLP syndrome. Although abnormal findings on MRI are almost always located only in the occipital and parietal lobe in most patients with non-associated PRES [13], many studies since then have shown that “atypical” variants abound [14], but the constellation of findings on imaging along with clinical features suggest PRES.

**Table 2:** Characteristics of PRES on MRI

Image conditions (MRI)	Vasogenic oedema	Cytotoxic oedema	PRES	Cerebral infraction (Acute stage)
FLAIR	↑	↑	↑	N, then ↑
DWI	N	↑	N	N, then ↑
ADC	↑	↓	↑	↓

*N* normal, *PRES* posterior reversible encephalopathy syndrome; ↑, hyperintense signal; ↓, hypointense signal

However, in HELLP syndrome, lesions are frequency observed in the brain stem, basal ganglia and thalamus, which are arterially supplied by small end arteries with little collateral circulation [15]. Based on these results, thrombotic microangiopathy in HELLP syndrome may result in higher incidence of those lesion [16]. However, FLIAR-MRI abnormalities in the occipital lobe, basal ganglia and callosal splenium were recognized in this case (Fig. 1a). This is a relatively rare case. Although the reversibility of vasogenic oedema in PRES is typical, early damage may also cause ischaemia [8]. PRES is usually considered a reversible condition if it is promptly diagnosed and properly treated. However, a delayed or incorrect diagnosis may lead to irreversible damage [17]. The differential diagnosis of PRES caused by stroke, cerebral venous thrombosis, encephalitis, and metabolic and demyelinating disorders may be difficult in pregnancy and puerperium [4]. In addition, given presence of basal ganglial and callosal lesions visualized on the MRI in this case (Fig. 1a), the differential diagnosis should be expanded to include hypoxic-ischaemic injury, or overdose of pain medication (e.g., opioids). The mistreatment of vasogenic oedema associated with PRES because ischaemic changes due to acute stroke might result in the stopping of prompt antihypertensive treatment. In contrast, the appropriate and rapid lowering of blood pressure in patients with PRES is essential to avoid any permanent brain injury, and PRES is reversible when early diagnosis is established and appropriate treatment is started without delay [18]. However, prospective studies are warranted to establish predictors of fatality in patients with PRES [19]. Therefore, MRI plays an important role in the prompt diagnosis of PRES.

In this case, corticosteroids were promptly used for the treatment of HELLP syndrome, and this treatment is considered to slow disease progression and prevent new major maternal morbidity [20]. Corticosteroids should reduce vasogenic oedema. Thus, aggressively used potent glucocorticoids are recommended for the management of patients considered to have HELLP syndrome [6]. Although there are only a few reports in the literature on the use of steroids in patients with PRES [21], corticosteroids might have been effective for PRES in this case.

## Conclusion

In this article, we report and highlight a rare association between severe HELLP syndrome and PRES. MRI findings were useful for the prompt diagnosis of PRES, which is characterized by hypersignals in FLAIR and ADC, but not in DWI. Additionally, the “atypical” MRI appearance of basal ganglial and callosal splenial involvement, as demonstrated in this case, may mistakenly lead clinicians to diagnose other aetiologies compared to typical PRES. It is considered that vasogenic oedema is the main pathology of PRES according to MRI image findings. This technique facilitated prompt diagnosis and treatment, resulting in a good outcome. We believe that clinicians should be aware of this neurological syndrome because prompt diagnosis and treatment may result in complete resolution.

## Abbreviations

ADC: Apparent diffusion coefficient
AST: Aspartate aminotransferase
Cr: Creatinine
DWI: Diffusion-weighted image
FLAIR: Fluid-attenuated inversion recovery
GCS: Glasgow coma scale
HELLP: Haemolysis, elevated liver enzymes, and low platelet count
LDH: Lactate dehydrogenase
MRI: Magnetic resonance imaging
PRES: Posterior reversible encephalopathy syndrome
